# Episodic memory in aging: neurocognitive bases and clinical implications of the distinction between hippocampal and frontal patterns

**DOI:** 10.1590/1980-5764-DN-2026-0505

**Published:** 2026-10-02

**Authors:** Pedro Donizetti de Oliveira

**Affiliations:** 1 Clínica Neuroavaliação e Neuroeducação, São Paulo SP, Brazil.

**Keywords:** Memory, Episodic, Aging, Cognitive Dysfunction, Hippocampus, Executive Function, Memória Episódica, Envelhecimento, Disfunção Cognitiva, Hipocampo, Função Executiva

## Abstract

Episodic memory is one of the cognitive domains most vulnerable to age-related changes and the early stages of neurodegenerative syndromes. In Brazilian clinical practice, however, the interpretation of memory performance frequently remains restricted to the quantitative analysis of delayed recall, without adequate consideration of the underlying neurocognitive mechanisms. This study aimed to critically examine the neurobiological foundations of the distinction between hippocampal consolidation deficits and executive retrieval impairments mediated by prefrontal circuits, discussing their diagnostic and gerontological implications. This integrative narrative review is grounded in recent national and international literature (2019–2024) and integrates evidence from neuroimaging, theoretical models of memory, and clinical data relevant to aging. Differentiating hippocampal consolidation deficits from executive retrieval impairments, integrated with the assessment of functional capacity, is proposed as a clinically actionable, mechanism-based framework for improving diagnostic accuracy in aging within heterogeneous sociocultural contexts such as Brazil.

## RESUMO.

A memória episódica é um dos domínios cognitivos mais vulneráveis às alterações relacionadas ao envelhecimento e aos estágios iniciais das síndromes neurodegenerativas. Na prática clínica brasileira, entretanto, a interpretação do desempenho mnemônico frequentemente permanece restrita à análise quantitativa da evocação tardia, sem a devida consideração dos mecanismos neurocognitivos subjacentes. Este estudo teve como objetivo examinar criticamente os fundamentos neurobiológicos da distinção entre déficits de consolidação hipocampal e prejuízos executivos de recuperação da informação mediados por circuitos pré-frontais, discutindo suas implicações diagnósticas e gerontológicas. Esta revisão narrativa integrativa fundamentase na literatura nacional e internacional recente (2019–2024) e integra evidências provenientes de estudos de neuroimagem, modelos teóricos da memória e dados clínicos relevantes para o envelhecimento. Propõe-se que a diferenciação entre déficits de consolidação hipocampal e prejuízos executivos de recuperação da informação, integrada à avaliação da capacidade funcional, constitua um modelo clínico aplicável, baseado em mecanismos, para aprimorar a acurácia diagnóstica no envelhecimento em contextos socioculturais heterogêneos, como o brasileiro.

## INTRODUCTION

 Brazil is experiencing an accelerated process of demographic transition, with substantial growth of the aged population in recent decades, accompanied by an increase in the demand for cognitive assessment at different levels of healthcare. This scenario imposes relevant challenges on health systems and gerontological practice, especially regarding the early identification of cognitive changes, the distinction between normative aging and pathological conditions, and the organization of care pathways in resource-limited settings^1^. Recent national studies have pointed to an increase in the prevalence of mild cognitive impairment and dementia in urban and rural contexts, reinforcing the need for more precise and clinically applicable interpretive models for the assessment of episodic memory in the country^1,2^. 

 In this context, subjective memory complaint is among the most frequent reasons for referral for neuropsychological assessment and specialized consultations. However, Brazilian evidence suggests that subjective complaint, in isolation, is not directly or consistently associated with structural impairment of the mnemonic system, being frequently modulated by emotional factors, educational level, stress, performance expectations, and characteristics of the sociocultural context. Brazilian educational heterogeneity, in particular, represents a critical variable: differences in schooling and educational quality affect performance on cognitive tasks and retrieval strategies, potentially increasing the likelihood of distorted interpretations when relying solely on quantitative score interpretation^3^. 

 Furthermore, gerontological practice requires that cognitive changes be interpreted in light of their actual impact on autonomy and functionality. Performance below expected levels on episodic memory tests does not always translate into significant functional impairment, especially in older adults with good cognitive reserve or effective environmental support^4^. Conversely, subtle and progressive functional changes may precede or accompany neurodegenerative cognitive decline, reinforcing the centrality of functional analysis in clinical reasoning. 

 In view of this reality, restricting the interpretation of episodic memory to isolated delayed recall becomes insufficient. Understanding the mechanisms underlying the observed failures — distinguishing, for example, hippocampal consolidation deficits from strategic retrieval inefficiencies associated with prefrontal circuits — contributes to greater diagnostic precision and reduces the risk of overdiagnosis in typical aging, especially in settings characterized by substantial sociocultural variability^5,6^. Thus, the need for integrative approaches that incorporate quantitative and qualitative performance, response to cues, recognition, and functionality is reinforced, supporting more ethical, contextualized, and mechanism-based gerontological practice^1,3^ . 

 This study aimed to critically examine, from a clinically grounded perspective, the neurobiological and functional distinction between hippocampal consolidation deficits and executive retrieval impairments, highlighting their implications for neuropsychological assessment in aging. In countries characterized by educational heterogeneity and unequal access to specialized diagnostic resources, such as Brazil, the adoption of mechanism-based interpretive models may represent a cost-effective strategy to enhance diagnostic precision. 

## METHODS

 This study consists of an integrative narrative review aimed at synthesizing contemporary evidence on episodic memory in aging, with emphasis on the distinction between hippocampal consolidation deficits and executive retrieval impairments. The literature search was conducted in the PubMed, Scopus, and SciELO databases, covering publications from January 2019 to December 2024. 

 The following keywords and MeSH terms were used in different combinations: “episodic memory,” “aging,” “hippocampus,” “prefrontal cortex,” “executive functions,” “memory consolidation,” and “cognitive impairment.” 

 Studies were selected based on their relevance to the neurocognitive mechanisms of episodic memory, clinical applicability to aging, and integration with neuroimaging or functional outcomes. Both original studies and review articles were considered. Articles not directly related to episodic memory mechanisms or lacking clinical applicability were excluded. 

 Given the narrative nature of this review, the selection process prioritized conceptual consistency, clinical relevance, and integration of findings rather than a systematic quantitative synthesis. 

## THEORETICAL MODELS OF EPISODIC MEMORY: CONTEMPORARY ADVANCES

 Classical multiple-system models established the distinction between episodic memory and semantic memory, attributing a central role in the formation and consolidation of contextualized autobiographical experiences to the medial temporal lobe, especially the hippocampus^7,8^. This perspective, widely established in cognitive neuropsychology, has led to the understanding that episodic memory constitutes a functionally distinct system, characterized by the conscious retrieval of events associated with temporal and spatial context^7,9-12^. 

 However, recent advances in structural and functional neuroimaging have expanded this conception by demonstrating that episodic memory does not depend exclusively on the hippocampus but rather on distributed and highly integrated networks^7,8^. Studies published between 2019 and 2024 demonstrate that episodic recall involves dynamic interactions among the medial temporal lobe, the dorsolateral prefrontal cortex, the posterior cingulate cortex, associative parietal regions, and executive control circuits^7,8^. From this perspective, episodic memory must be understood as a product of functional connectivity between consolidation systems and cognitive control systems. 

 Contemporary models of systems consolidation suggest that the hippocampus acts as an indexing structure, coordinating the reactivation of distributed cortical representations^9^. The effective retrieval of these representations depends on the integrity of strategic search and monitoring mechanisms mediated by the prefrontal cortex^8^. Thus, free recall requires not only adequate storage but also the capacity to organize internal cues, inhibit interference, and select relevant responses^8^. 

 This integrative approach explains why similar behavioral manifestations — such as reduced performance on delayed recall — can result from distinct mechanisms^5,6^. Impairment may stem from hippocampal consolidation deficits, characteristic of early neurodegenerative conditions, or from inefficient strategic retrieval, frequently associated with normative age-related executive changes^5,6^. The distinction between these mechanisms is not merely theoretical but has direct implications for differential diagnosis. 

 Additionally, recent studies emphasize the role of functional connectivity between the hippocampus and the prefrontal cortex in maintaining mnemonic performance throughout aging^4^. Understanding the functional architecture underlying cognitive performance constitutes an indispensable element of accurate clinical interpretation. The analysis of episodic memory must therefore transcend the isolated interpretation of scores and incorporate an understanding of the neural systems that support consolidation and retrieval. 

## HIPPOCAMPAL CONSOLIDATION: RECENT EVIDENCE

 The consolidation of episodic memory depends critically on the integrity of the hippocampus and adjacent structures of the medial temporal lobe, including the entorhinal cortex, parahippocampal gyrus, and Papez circuit^5,10^. Recent structural and functional neuroimaging studies have demonstrated that changes in these regions frequently precede more evident clinical manifestations of Alzheimer disease, constituting some of the earliest biological markers of the neurodegenerative process^5,10^. 

 From a neurobiological standpoint, consolidation involves processes of synaptic stabilization and systems-level integration of mnemonic traces distributed throughout the associative cortex^9^. Hippocampal dysfunction compromises this integration, resulting in unstable or incomplete traces, which clinically manifest as an accelerated rate of forgetting and a lack of significant benefit from structured cues^9^. Unlike predominantly executive deficits, recognition also tends to be impaired in these cases, suggesting a failure of storage itself^9^. 

 Longitudinal studies have shown that the absence of improvement with semantic cues or forced recognition constitutes a behavioral marker sensitive to early hippocampal dysfunction^6^. In individuals with amnestic mild cognitive impairment, a pattern characterized by reduced recall, impaired recognition, and low responsiveness to cues is frequently observed — a profile that correlates with a higher risk of conversion to Alzheimer disease during clinical follow-up^5,6^. 

 In the Brazilian context, investigations published in journals such as Dementia & Neuropsychologia and *Arquivos de Neuro-Psiquiatria* (2019–2024) have highlighted the relevance of the qualitative assessment of episodic consolidation in clinical practice^1,2^. These publications emphasize that the isolated interpretation of free recall may be insufficient, making it essential to consider the learning pattern, rate of forgetting, and response to cues to more accurately infer hippocampal integrity. National guidelines for the diagnosis of dementia reinforce the importance of integrating clinical, functional, and cognitive data in the characterization of mnemonic impairment, thereby avoiding both underdiagnosis and overinterpretation of changes compatible with normative aging^2^. 

## EXECUTIVE RETRIEVAL AND AGING

 Fronto-subcortical changes associated with typical aging affect the efficiency of strategic search and monitoring of stored information^8,11^. Unlike hippocampal impairment, which affects the consolidation of the mnemonic trace, the executive changes observed in normative aging tend to compromise primarily the retrieval stage, especially in tasks that require free recall without external support^8, 11^. 

 The dorsolateral and ventrolateral prefrontal cortex play a central role in the organization of encoding, the selection of internal cues, the inhibition of irrelevant responses, and the monitoring of recall accuracy^8^. With advancing age, a gradual reduction in the efficiency of these functions is observed, frequently associated with changes in connectivity between frontal regions and subcortical structures, such as the basal ganglia and thalamus^4^. 

 Recent research demonstrates that healthy older adults show greater benefit when memory tasks provide structured semantic cues or recognition alternatives, indicating that the mnemonic trace has been adequately stored but that spontaneous access is less efficient^7^. This behavioral pattern is compatible with reduced executive control efficiency rather than structural impairment of the consolidation system. Furthermore, functional neuroimaging studies indicate that, in typical aging, compensatory recruitment of additional prefrontal regions may occur during episodic memory tasks, preserving overall performance, albeit at the cost of greater cognitive effort^4^. 

 In the clinical context, executive retrieval impairment is characterized by reduced recall, consistent improvement with cues, and preserved recognition^7,8^. The rate of forgetting tends to remain stable over time, unlike the pattern observed in neurodegenerative conditions, and the preservation of everyday functionality constitutes a relevant indicator that the observed impairment is situated predominantly in the executive domain. 

 In the Brazilian context, educational heterogeneity can amplify differences in the use of retrieval strategies^3^. Individuals with less formal education may show less spontaneity in organizing material during encoding, increasing their dependence on structured cues. Thus, the interpretation of executive impairment must consider sociocultural variables, avoiding the automatic attribution of pathological meaning to patterns compatible with normative aging. 

## HIPPOCAMPAL–PREFRONTAL CONNECTIVITY IN AGING

 Recent research has emphasized that episodic memory cannot be understood exclusively from the perspective of isolated structures but must be analyzed within the framework of distributed and dynamically integrated functional networks^7,8^. Functional neuroimaging studies demonstrate that episodic recall involves coordinated interactions among the medial temporal lobe, the dorsolateral and ventromedial prefrontal cortex, the posterior cingulate cortex, and associative parietal regions, forming broad networks related to the contextualized retrieval of experience^7,8^. 

 Connectivity between the hippocampus and the prefrontal cortex is particularly relevant to strategic retrieval. The hippocampus acts as an index that reactivates distributed representations, whereas the prefrontal cortex organizes the search, selects internal cues, and monitors response accuracy^8^. When synchrony between these regions is reduced, even in the absence of significant structural atrophy, a spontaneous recall may become less fluent^4^. 

 In typical aging, a gradual reduction in the efficiency of this connectivity is observed, especially in executive and cognitive control networks, whereas hippocampal structural integrity may remain relatively preserved during the early stages^4^. In contrast, in early Alzheimer disease, hippocampal volumetric reduction associated with decreased connectivity with parietal and frontal regions results in failure of the formation or stabilization of the episodic trace, making recall impaired even in the presence of structured cues and recognition significantly compromised^5,10^ . 

 Understanding episodic memory as a product of the interaction between hippocampal-prefrontal networks allows reductionist interpretations to be overcome and supports more refined clinical assessments. The integration of evidence from connectivity, behavioral patterns, and functional impact constitutes a solid basis for differentiating normative aging from emerging pathological processes. 

## NORMAL AGING, MILD COGNITIVE IMPAIRMENT, AND ALZHEIMER DISEASE: CONTINUITY AND DISRUPTION

 Cognitive aging must be understood as a heterogeneous process influenced by biological, educational, psychosocial, and contextual factors^4^. Although subtle changes in executive functions, processing speed, and spontaneous recall efficiency are frequently observed in typical aging, the maintenance of functional autonomy and adaptative capacity constitutes a fundamental criterion for its characterization. Minor difficulties with free recall do not necessarily imply structural impairment of the mnemonic system. 

 Amnestic mild cognitive impairment (aMCI) occupies an intermediate position between typical aging and established dementia^1,6^ and is characterized by lower-than-expected performance for age and educational attainment on episodic memory tasks, with relative preservation of functional independence. However, this diagnostic category is not homogeneous: some individuals remain stable or show improvement over time, whereas others progress to Alzheimer disease^6^. Differentiating between these trajectories depends on the qualitative analysis of the mnemonic pattern, including the rate of forgetting, recognition, and response to cues^5,6^ . 

 In early Alzheimer disease, a qualitative disruption in the pattern of performance is observed^5,10^. The rate of forgetting becomes accelerated, recognition is impaired, and the absence of benefit from cues indicates primary hippocampal consolidation failure. Concurrently, impairments in instrumental activities of daily living begin to emerge, reflecting progressive functional impact. Differentiating normative continuity from pathological disruption requires a mechanistic analysis of performance rather than mere statistical classification based on standard deviations. 

## NEUROPSYCHOLOGICAL DISSOCIATION: EVIDENCE APPLIED TO BRAZILIAN PRACTICE

 In the Brazilian context, factors such as heterogeneous educational attainment, socioeconomic inequality, cultural diversity, and unequal access to specialized services significantly influence performance on neuropsychological tests^3^. Exclusive reliance on standardized norms, often based on samples with educational profile that differ from those of the population being served, can lead to distorted interpretations and inappropriate classifications. 

 In this setting, qualitative analysis assumes a central role. Elements such as the learning curve, patterns of intrusions, perseverations, organization strategies during encoding, and response to cues provide richer information about the mechanisms underlying performance^11^. Consistent improvement with semantic or phonemic cues suggests preserved storage and points to strategic retrieval inefficiency, frequently associated with normative aging or mild executive changes^8,11^. In contrast, the absence of improvement with cues and impaired recognition indicate potential consolidation failure, warranting more in-depth investigation^5,10^. 

 Recent national research reinforces that integrating cognitive performance with functional assessment significantly improves diagnostic accuracy, especially in public health services and primary care settings. The dissociative model offers a rational framework for clinical decision-making, even when advanced diagnostic resources are unavailable, contributing to assessments that are more ethical, contextualized, and aligned with the needs of the Brazilian older adult population. 

## RECOGNITION AS A DIFFERENTIAL MARKER IN AGING

 Recognition, frequently undervalued in clinical practice compared with free recall, constitutes an essential component in distinguishing retrieval failure from consolidation failure^9^. Whereas spontaneous recall depends heavily on executive mechanisms and strategic search, recognition involves greater external support, reducing the demand on executive control and allowing for a more direct assessment of the integrity of the stored mnemonic trace^9^. 

 When recognition remains preserved, even in the presence of reduced recall, it can be inferred that the mnemonic trace was adequately consolidated and that the difficulty lies predominantly at the stage of strategic access, frequently associated with fronto-subcortical changes or normative age-related changes^8^. In contrast, significant impairment in recognition, especially when accompanied by low responsiveness to structured cues and an accelerated rate of forgetting, suggests impairment of the consolidation system^5,10^. 

 Recent clinical evidence has reinforced the value of structured paradigms such as the Free and Cued Selective Reminding Test (FCSRT) in operationalizing this dissociation. By ensuring controlled encoding through categorical cues and subsequently evaluating recall with and without those cues, the FCSRT enables a systematic distinction between retrieval-based difficulties and genuine storage failures. In individuals whose episodic memory impairment is primarily executive in nature, semantic cueing normalizes recall, reflecting intact consolidation; in contrast, patients with hippocampal dysfunction show limited or absent benefit from cues, indicative of a primary storage deficit^13 ,14^. 

 Building on this paradigm, the Staging of Objective Memory Impairment (SOMI) framework was developed to characterize the sequential progression of mnemonic decline across the Alzheimer disease continuum. SOMI stages 1–2 are defined by reductions in free recall that remain remediable with cueing, reflecting retrieval inefficiency with preserved storage; stages 3–4 are characterized by failure to benefit from cues and impaired total recall, marking the transition to a consolidation deficit consistent with incipient neurodegeneration^14,15^. This staging system has demonstrated predictive validity in longitudinal cohorts, with impaired free recall preceding dementia diagnosis by approximately seven years and failure to benefit from cues by approximately two to four years^15^. A recent systematic review published in Dementia & Neuropsychologia further confirmed the clinical validity of the FCSRT and its correlations with biomarkers in diverse populations, including its potential applicability in Latin American contexts^13^. 

 In the clinical context, the integrated analysis of recall, recognition, and response to cues allows the differentiation of profiles that are apparently similar from a quantitative perspective. Thus, recognition ceases to be a complementary component and becomes central to the mechanistic interpretation of mnemonic performance. 

## EPISODIC MEMORY, FUNCTIONAL CAPACITY, AND GERONTOLOGY

 Contemporary gerontology emphasizes functional capacity as the organizing principle of healthy aging, shifting the exclusive focus from nosological diagnosis toward the maintenance of autonomy, social participation, and quality of life. In this context, cognitive changes must be interpreted in light of their actual impact on the daily lives of older adults. 

 Episodic memory plays a fundamental role in the organization of daily routine, directly influencing activities such as financial management, medication administration, appointment planning, and decision-making. However, mild executive deficits or difficulties with spontaneous recall can coexist with substantial functional preservation, especially in individuals with high cognitive reserve or well-established compensatory strategies^4^. 

 Neuropsychological assessment should therefore integrate cognitive and functional data within a contextualized analysis. Instruments that assess instrumental activities of daily living, when interpreted together with qualitative patterns of episodic memory, provide a more comprehensive picture of the actual impact of the observed changes. Considering the psychosocial context — including family support, educational attainment, and environmental demands — is essential to avoid the excessive medicalization of changes compatible with normative aging^3^. 

## CRITICAL DISCUSSION

 The distinction between consolidation and retrieval represents a relevant conceptual advance in the understanding of episodic memory in aging. However, its clinical application requires caution and contextualization. Episodic memory involves distributed networks and dynamic interactions between medial temporal systems and executive circuits; therefore, behavioral patterns should not be interpreted in a simplistic or binary manner^7,8^. 

 In the Brazilian context, characterized by broad sociocultural heterogeneity, the adoption of an integrative model contributes to reducing the risk of overdiagnosis and aligning neuropsychological practice with the ethical principles of contemporary gerontology^3^. The exclusively quantitative interpretation of scores can lead to the inappropriate labeling of older adults with typical aging, generating anxiety, stigmatization, and unnecessary interventions. 

 Conversely, underestimating consistent signs of consolidation failure — such as the absence of benefit from cues and impaired recognition — can delay timely diagnosis and compromise appropriate therapeutic planning^5 ,10^. Interpretive balance is grounded in the mechanistic understanding of performance, the qualitative analysis of the mnemonic pattern, and the integration of functional and contextual data. 

 From a clinical standpoint, this distinction can be illustrated by two profiles frequently encountered in neuropsychological practice. An older adult presenting with reduced free recall but meaningful improvement with semantic cues, intact recognition, and preserved daily functioning is more consistent with executive retrieval inefficiency associated with normative aging. In contrast, a patient showing reduced recall, absent or minimal benefit from cueing, impaired recognition, and emerging difficulties in instrumental activities of daily living raises clinical concern for hippocampal consolidation failure and possible early neurodegeneration (Figure 1). This qualitative differentiation, grounded in the mechanistic framework presented throughout this review, supports more precise, ethically responsible, and individualized clinical decision-making in gerontological practice. 

**Figure 1:**
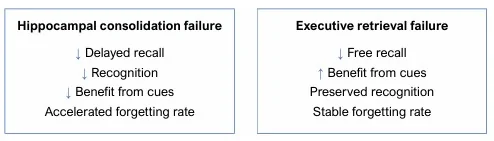
Dissociation between hippocampal and executive dysfunction patterns in aging.

 Thus, the proposed model should not be understood as a rigid diagnostic formula but as an interpretive framework that organizes clinical reasoning and supports evidence-based, mechanism-based decision-making. 

## LIMITATIONS AND BRAZILIAN RESEARCH AGENDA

 Although recent international advances in biomarker-based frameworks have reshaped the conceptualization of Alzheimer disease^10^, their routine applicability in low- and middle-income countries remains limited. The empirical validation of the integrative model presented here in Brazilian samples is still incipient, as most evidence on functional connectivity and biomarkers is derived from international studies conducted in distinct sociocultural contexts^7,10^. 

 Longitudinal studies are particularly relevant for correlating response to cues and recognition performance with structural biomarkers, such as hippocampal volumetry, and with ecological measures of functioning^5,10^. Future research should also incorporate ecological measures of functioning and consider sociocultural variables such as educational attainment, educational quality, and access to cognitive stimulation throughout life^3^. The incorporation of these dimensions will allow the development of interpretive models that are more sensitive to the Brazilian context, strengthening national scientific production in gerontology and the neuropsychology of aging. 

 In conclusion, the interpretation of episodic memory in aging requires moving beyond strictly quantitative approaches and incorporating a mechanistic perspective grounded in robust neurocognitive foundations. The distinction between hippocampal consolidation deficits and strategic retrieval deficits associated with prefrontal circuits is not merely a conceptual distinction but rather a fundamental framework for sound clinical reasoning in contemporary gerontological practice. 

 In the Brazilian context, characterized by educational heterogeneity, sociocultural inequalities, and a high prevalence of subjective memory complaints, the isolated analysis of delayed recall is insufficient to support consistent diagnostic inferences. Integrating recall patterns, response to cues, recognition performance, and functional assessment allows a more precise understanding of the predominantly affected neurocognitive system, reducing the risk of overdiagnosing neurodegenerative conditions in older adults with typical aging. 

 The integration of episodic memory and functional capacity reinforces the alignment of the proposed model with the principles of contemporary gerontology, which prioritize autonomy, social participation, and quality of life. Functional preservation, even in the presence of subtle executive deficits, must be considered a central element of clinical interpretation, avoiding the excessive medicalization of changes compatible with normative aging. 

 From a scientific standpoint, the integrative model discussed herein contributes to bridging evidence from neuroimaging, contemporary theories of memory consolidation, and clinical data applicable to the Brazilian context. By emphasizing the distinction between storage and retrieval, it promotes a more refined, ethically responsible, and mechanism-based neuropsychological assessment aligned with evidence-based practice. Furthermore, by operationalizing the dissociation between consolidation and retrieval within routine neuropsychological assessment, this framework offers a pragmatic and scalable model adaptable to diverse healthcare settings. 

 By incorporating the qualitative analysis of recall, cueing, and recognition patterns into routine clinical assessment, neuropsychology can move beyond purely quantitative interpretations and achieve greater diagnostic precision in aging. 

## Data Availability

No new data were generated or analyzed in this study.
